# Clinical and Demographic Profile of Patients Undergoing Surgery for Chronic Subdural Hematoma at a Tertiary Care Hospital in Nepal: An Observational Study

**DOI:** 10.31729/jnma.9070

**Published:** 2025-06-30

**Authors:** Rupesh Raut, Himal Karki, Dinuj Shrestha, Dipendra Chapagain, Jay Bhusan Jha, Sushant Regmi, Prakash Bista

**Affiliations:** 1 Department of Neurosurgery, Patan Academy of Health Sciences, Lagankhel, Lalitpur, Nepal; 2 Patan Academy of Health Sciences, Lagankhel, Lalitpur, Nepal; 3 Devchuli Municipal Hospital, Devchuli, Nawalparasi East, Nepal; 4 Department of General Practice and Emergency Medicine, Patan Academy of Health Sciences, Lagankhel, Lalitpur, Nepal

**Keywords:** *chronic subdural hematoma*, *clinical profile*, *mortality*, *Nepal*, *neurosurgery*

## Abstract

**Introduction:**

Chronic subdural hematoma is increasingly common in aging populations and presents with a wide range of presentations. Despite its rising incidence, there is a lack of clinical and demographic characterization of chornic subdural hematoma in the Nepali population. This study aimed to describe the demographic and clinical characteristics of chornic subdural hematoma patients.

**Methods:**

A retrospective observational cross-sectional study was conducted in the Department of Neurosurgery of a tertiary care center. Data were retrospectively collected from all patients who underwent surgery for chornic subdural hematoma between March 2022 and March 2024. Variables such as age, sex, clinical presentation, risk factors, length of hospital stay, and in-hospital mortality were recorded. Data were entered into Microsoft Excel and analyzed using the Statistical Package for Social Sciences software 16.0.

**Results:**

Among 52 patients, 39 (75%) were male. The mean age of patients was 63.10±14.72 years (95% CI: 59.00-67.21), with 34 (65.38%) patients aged over 60 years. The median length of hospital stay was 7 days (IQR: 6-8 days). Twenty-five (48.08%) patients had a history of head trauma, followed by 15 (28.85%) with chronic alcohol use. Motor weakness, altered mental status, and headache were common clinical manifestations, presenting in 27 (51.92%), 18 (34.62%), and 15 (28.85%) patients, respectively. There were 4 (7.69%) recurrences and 3 (5.77%) deaths following surgery for chornic subdural hematoma.

**Conclusions:**

Chornic subdural hematoma was more common among elderly male, especially those with a history of head trauma and chronic alcohol use. Motor weakness and unilateral hematomas were common findings.

## INTRODUCTION

Chronic subdural hematoma (CSDH) is one of the common neurosurgical conditions presenting with mild symptoms to coma.^1^ Globally, the overall incidence of CSDH ranges 1.7-20.6 per 100,000 persons per year, and it is rising.^2^ Risk factors for CSDH include advanced age, male gender and use of anticoagulant or antiplatelet drugs.^3^ Burr hole craniotomy is commonly used surgical management for CSDH, showing the favorable outcomes globally.^2,4^ CSDH has a variety of outcomes including recurrence rate ranging 10-20% and mortality rate ranging up to 32%.^2^

Studies from US and UK projected an increase in CSDH incidence to 121.4 and 150 cases per 100,000 persons respectively by 2030.^5,6^ Despite the rising incidence of CSDH, there is a lack of clinical and demographic characterization of patients with CSDH in Nepal which is essential to neurosurgeons for approaching and managing such conditions.^7^ This study aimed to describe the clinical and demographic profile of the patients undergoing surgery for chronic subdural hematoma in a tertiary health care center in Nepal.

## METHODS

This retrospective, observational, cross-sectional study was conducted at the newly established Department of Neurosurgery of a tertiary care hospital in Nepal. The study period covered two years following the establishment of the neurosurgery department, from March 2022 to March 2024. All the patients who were diagnosed and operated on for CSDH during the study period were included in the analysis. A census sampling method was employed, including all eligible patients diagnosed and operated on for CSDH during the study period.

Data were collected from the medical records and discharge summaries of patients diagnosed and operated for CSDH using a structured proforma to extract clinical and demographic information. Variables such as age and sex were collected to explain the demographic characteristics of CSDH patients. Clinical characteristics of the patients were captured with variables such as length of hospital stay, clinical presentation (headache, nausea, vomiting, motor weakness, altered consciousness, seizures and speech disturbances), known risk factors (recent head trauma, chronic alcoholism, known seizure disorder, use of anticoagulants or antiplatelet drugs, coagulopathies and previous shunt surgery), recurrence after previous surgery, and in-hospital mortality. Radiological findings (laterality of hematoma and presence of midline shift) on either computed tomography of the head (CT head) or magnetic resonance imaging of the brain (MRI brain) were also collected. Data points with missing values on any of the following variables: length of hospital stay, clinical presentation, known risk factors, recurrence, and in-hospital mortality were excluded from the analysis.

In this study, motor weakness was considered if there was hemiparesis, difficulty or inability to walk excluding the generalized body weakness as well as bowel/bladder incontinence. Altered mental status was considered a clinical presentation if there was decreased response or consciousness, drowsiness, loss of consciousness, decreased sensorium and abnormal talk excluding dizziness.

Data were entered on a Microsoft Excel spreadsheet and exported to Statistical analysis was performed using SPSS Statistics for Windows, version 16.0 (SPSS Inc., Chicago, Ill., USA) for statistical analysis. Descriptive statistics were used to summarize the data. Means and standard deviations used for continuous variables, and frequencies and percentages used for categorical variables.

Patient identifiers were removed and replaced with unique study codes during data extraction to ensure the confidentiality of the patients. The anonymized dataset was stored in a password-protected computer accessible only to the research team. No personal identifiers were used in data presentation or analysis. Ethical approval for the study was obtained from the Institutional Review Committee of Patan Academy of Health Sciences (IRC-PAHS), Lalitpur, Nepal (Reference No: drs2404191848) and data collection was started afterwards. As this was a retrospective study,the requirement for individual patient consent was waived by the IRC.

## RESULTS

A total of 52 patients diagnosed with CSDH were operated on during the study period. Data for all variables were available for all 52 patients, except for radiological findings, which were available for 49 patients. The mean age of the patients was 63.10 ± 14.72 years (95% CI: 59.00-67.21). There were 39 (75%) male patients, with a male-to-female ratio of 3:1. The median length of hospital stay was 7 days (IQR: 6-8 days), with minimum stay of 2 days and a maximum stay of 56 days. Thirty-four (65.38%) patients were over 60 years of age, while 5 (9.62%) were aged 18-39 years (Table 1).

**Table 1:** Demographic characteristics of patients diagnosed with chronic subdural hematoma (n=52).

Characteristics	n(%)
Sex	
Male	39(75)
Female	13(25)
**Age group**	
18-39 years	5(9.62)
40-59 years	13(25)
60 years or more	34(65.38)

Among 52 patients, 25 (48.08%) had a history of recent head trauma. This was followed by 15 (28.85%) patients with chronic alcohol use and 3 (5.77%) had known seizure disorder. One patient (1.92%) each was taking antiplatelet drugs, had previous shunt surgery, or had coagulopathy.

Motor weakness, altered mental status, and headache were most common clinical presentations, occurring in 27 (51.92%), 18 (34.62%) and 15 (28.85%) patients, respectively. Nine patients (17.31%) presented with vomiting, six (11.54%) with speech disturbances, four (7.69%) with nausea, and 2 (3.85%) with seizures (Table 2).

**Table 2:** Risk factors and clinical presentation of patients diagnosed with chronic subdural hematoma (n=52).

Characteristics	n(%)
**Risk factors**	
Recent head trauma	25(48.08)
Chronic alcoholism	15(28.85)
Known seizure disorder	3(5.77)
Antiplatelet	1(1.92)
Coagulopathy	1(1.92)
Previous shunt surgery	1(1.92)
Clinical presentation	
Motor weakness	27(51.92)
Altered mental status	18(34.62)
Headache	15(28.85)
Vomiting	9(17.31)
Speech disturbances	6(11.54)
Nausea	4(7.69)
Seizure	2(3.85)

Among the 49 patients with available neuroimaging, 44 (89.8%) had unilateral hematomas, while 5 (10.2%) had bilateral hematomas (Table 3). Twelve (24.49%) patients had midline shift. Among unilateral hematomas, 24 (54.55%) patients had left-sided hematomas, and 20 (45.45%) patients had right-sided hematomas. Among the 52 patients, 4 (7.69%) were admitted for recurrent hematoma after surgery. There were 3 (5.77%) inhospital mortalities following surgery.

**Table 3:** Radiographic findings of patients diagnosed with chronic subdural hematoma (n=49).

Characteristics	n(%)
Unilateral hematoma	44(89.80)
Left-sided hematoma	24(54.55)
Right-sided hematoma	20(45.45)
Bilateral hematoma	5(10.20)
Midline shift	12(24.49)

## DISCUSSION

This study assessed the clinical and demographic characteristics of patients with CSDH, contributing to the growing literature on this condition in low-resource settings. The mean age of patients in our study was 63.1 years, with a predominance of male sex. This finding aligns with a previous study conducted in Nepal, which reported a mean age of 63.8 years and a male proportion of 85%.^8^ Similarly, a study done in China by Ou et. al also reported a mean age of 63.1 years with 82.6% of patients being male.^9^ In contrast, studies from the UK and USA reported a higher mean patient age of 80.6 years and 83.8 years, respectively.^10,11^

CSDH involves the accumulation of blood products within the subdural space and typically follows an insidious, indolent course.^11^ The increased vulnerability of bridging veins to tear in old age and age-related atrophy, predisposes the elderly patient to hematoma formation and CSDH development.^3^ Additionally, advanced age and male gender are well-noted and established risk factors for the development of CSDH.^3^

Similar to a retrospective cohort study that documented 59.1% of CSDH patients had traumatic etiology, our study also reported history of head trauma as a common etiology, present in 48.08% of patients.^1^ A similar study from Nepal showed that 53.8% of CSDH patients had a history of recent head trauma.^4^ In our study, more CSDH patients were chronic alcohol consumers than anticoagulant or antiplatelet users (28.85% versus 1.92%). In contrast to the Western countries where anticoagulant and antiplatelet therapy is frequent among CSDH patients.^2^ For instance, Jin Eun et al. documented anticoagulant therapy in 25.76% of CSDH patients undergoing surgery.^12^ This discrepancy is likely due to the higher prevalence of alcohol use and alcohol-related fall injuries in Nepal.^13^ In addition to that, Sedain et al. identified alcohol abuse as a key contributor to the development of CSDH in the Nepali population.^14^

In our study, motor weakness and altered mental status were common clinical presentations, occurring in 51.92% and 34.62% of patients, respectively. A previous study done by Bartek J et al. reported limb weakness in 44.8% of the elderly CSDH patients.^15^ However, other studies have documented the headache as a common clinical presentation in CSDH patients, with reported frequencies ranging from 38% to 90%, which is higher than the 28.85% observed in our study.^4,9,15-18^ This variation may be attributed to differences in symptom perception and reporting, particularly among elderly patients who frequently have cognitive impairment. CSDH in the elderly often presents with altered mental status, manifest as confusion, drowsiness, but can also present with hemiparesis, ataxia, vertigo, nystagmus, epilepsy, and in more serious cases, even coma.^19^ Besides that, elderly patients are less likely to present with headache possibly due to the reduced mass effect of hematoma on the atrophied brain of elderly people.^15^

Our study found that majority of the patients (89.80%) had unilateral hematoma, consistent with findings from other studies.^1,4^ In contrast, other studies reported the bilateral hematomas in patients ranging from 15% to 22%, whereas bilateral hematomas was present in only 10.20% of the patients in our study.^8,20^ Bilateral are associated with higher recurrence rates of CSDH, prolonged hospital stays, and increased mortality rates.^21,22^

At our center, we only do single burr hole with thorough washing without postoperative drain placement. A survey among Nepali neurosurgeon reported 74% preferred single burr hole with 100% surgeons washed the subdural space and 36% did not place the drain.^14^ Mortality rate following surgery in our study was 5.77%, which is lower than the rates reported by other studies (ranging from 10% to 32%).^2,23,24^ Additionally, the recurrence rate of CSDH was also lower (7.69%) in our study than the global average of 10% to 20%, possibly due to lower bilateral hematoma presentation.^2^ However, findings should be interpreted with caution due to small sample size (n=52) and lack of long-term follow-up in our study.

One of the key strengths of this study is its contribution to the limited literature on CSDH in the Nepali population. This study provides valuable regional data from a health system that is often underrepresented in the global neurosurgical literature. Such insights are crucial for understanding how CSDH presents in resource-limited settings. The study findings highlight the importance of early identification and intervention in elderly patients presenting with neurocognitive or motor symptoms, especially those with a history of head trauma or alcohol use. Furthermore, this study emphasizes the importance of local data in understanding region- specific risk factors, clinical patterns, and outcomes, which may differ from those reported in high-income countries.

However, there are several important limitations to consider when interpreting the findings of this study. Firstly, the relatively small sample size, combined with the retrospective nature of the research, inherently restricts the ability to generalize the results to broader populations. The limited number of cases may not capture the full clinical variability seen in patients with chronic subdural hematoma (CSDH), and retrospective data collection is subject to the risk of missing or incomplete information. For example, the lack of comprehensive documentation on anticoagulant or antiplatelet use may have led to potential inaccuracies in identifying key risk factors, thereby influencing the overall analysis and conclusions. Furthermore, since the study was conducted at a newly established neurosurgical center, there is a possibility of selection bias, as the center may have only received a specific subset of patients, such as those with more severe presentations or those referred for surgical intervention, limiting the representativeness of the general population affected by CSDH. Also, this study did not assess the long-term outcomes, functional recovery after surgery, and postoperative complications, limiting the understanding of recovery after surgery and the prognosis of CSDH.

Future research should focus on conducting large- scale, multicenter, prospective studies to improve the reliability and applicability of the findings. Such studies would help validate these initial observations and provide deeper insights into long-term outcomes, including postoperative morbidity, recurrence, and mortality, thereby offering a more comprehensive understanding of the surgical management of chronic subdural hematoma.

## CONCLUSIONS

This study showed that CSDH remains a significant neurosurgical condition in elderly populations, particularly affecting males with risk factors such as recent head trauma and chronic alcohol use. The common clinical presentations were motor weakness, altered mental status, and headache which were consistent with global patterns. Unilateral hematomas were more common than bilateral ones.
